# Cefepime and New Cefepime/Beta-Lactamase Inhibitor Combination for the Treatment of Gram-Negative Bacteria: Chemical Structure and Mechanism of Action, Microbiological Target, Clinical Use and PK/PD Characteristics

**DOI:** 10.3390/ph19020283

**Published:** 2026-02-07

**Authors:** Davide Carcione, Gioconda Brigante, Antonella Carducci, Jari Intra, Simone Ambretti, Floriana Campanile, Gabriele Arcari, Nicasio Mancini, Dario Cattaneo, Floriana Gona, Mariagrazia Perilli, Alessandra Piccirilli, Nicholas Geremia, Verena Zerbato, Stefano Di Bella, Giovanna Maria Nicolò, Luigi Principe

**Affiliations:** 1 Laboratory of Clinical Microbiology and Virology, ASST Valle Olona, 21013 Gallarate, VA, Italy; gioconda.brigante@asst-valleolona.it (G.B.); antonella.carducci@asst-valleolona.it (A.C.); 2 Clinical Chemistry Laboratory, Fondazione IRCCS San Gerardo Dei Tintori, 20900 Monza, Italy; jari.intra@irccs-sangerardo.it; 3 Microbiology Unit, IRCCS Azienda Ospedaliero-Universitaria of Bologna, 40138 Bologna, Italy; simone.ambretti@aosp.bo.it; 4 Department of Medical and Surgical Sciences, Alma Mater Studiorum, University of Bologna, 40138 Bologna, Italy; 5 Department of Biomedical and Biotechnological Sciences, Section of Microbiology, University of Catania, 95123 Catania, Italy; f.campanile@unict.it; 6 Laboratory of Medical Microbiology and Virology, Department of Medicine and Technological Innovation, University of Insubria, 21100 Varese, Italy; gabriele.arcari@uninsubria.it (G.A.); nicasio.mancini@uninsubria.it (N.M.); 7 Laboratory of Medical Microbiology and Virology, Fondazione Macchi University Hospital, 21100 Varese, Italy; 8 Department of Biomedical Sciences, Humanitas University, Pieve Emanuele, 20072 MI, Italy; dario.cattaneo@hunimed.eu; 9 Department of Biomedical and Biotechnological Sciences (BIOMETEC), University of Catania, 95123 Catania, Italy; floriana.gona@unict.it; 10 Department of Biotechnological and Applied Clinical Sciences, University of L’Aquila, 67100 L’Aquila, Italy; mariagrazia.perilli@univaq.it (M.P.); alessandra.piccirilli@univaq.it (A.P.); 11 Unit of Infectious Diseases, Department of Clinical Medicine, Ospedale “dell’Angelo”, 30174 Venice, Italy; nich.geremia@gmail.com; 12 Unit of Infectious Diseases, Department of Clinical Medicine, Ospedale Civile “S.S. Giovanni e Paolo”, 30122 Venice, Italy; 13 Infectious Disease Unit, Trieste University Hospital (ASUGI), 34125 Trieste, Italy; verena.zerbato@gmail.com (V.Z.); stefano932@gmail.com (S.D.B.); 14 Clinical Department of Medical, Surgical, and Health Sciences, Trieste University, 34129 Trieste, Italy; 15 Microbiology and Virology Unit, Great Metropolitan Hospital “Bianchi-Melacrino-Morelli”, 89100 Reggio Calabria, Italy; giovannamaria.nicolo@ospedalerc.it (G.M.N.); luigi.principe@gmail.com (L.P.)

**Keywords:** cefepime, β-lactam/β-lactamase inhibitor (BL/BLI) combinations, zidebactam, taniborbactam, enmetazobactam, nacubactam, PK/PD features

## Abstract

The global spread of multidrug-resistant (MDR) Gram-negative bacteria, particularly extended-spectrum β-lactamase (ESBL)- and carbapenemase-producing *Enterobacterales*, *Pseudomonas aeruginosa*, and *Acinetobacter baumannii*, presents a significant public health challenge by limiting effective antimicrobial treatment options. Cefepime, a fourth-generation cephalosporin with broad-spectrum activity, is increasingly compromised by β-lactamase production, efflux pumps, and porin loss. In response, novel cefepime-based β-lactam/β-lactamase inhibitor (BL/BLI) combinations have been developed to overcome these resistance mechanisms. This review examines preclinical and clinical studies on cefepime-based BL/BLI combinations, specifically cefepime/enmetazobactam, cefepime/taniborbactam, cefepime/zidebactam, and cefepime/nacubactam, as found in the PubMed database. Key findings include the restoration of activity against class A ESBLs with cefepime/enmetazobactam, while cefepime/taniborbactam and cefepime/zidebactam show broader inhibition of serine β-lactamases and selected metallo-β-lactamases. Additionally, zidebactam and nacubactam target penicillin-binding protein 2, enhancing bactericidal potency. Preclinical and early-phase clinical trial data indicate potent in vitro activity and favorable pharmacokinetic/pharmacodynamic (PK/PD) profiles. Specifically, the combination of cefepime with enmetazobactam has demonstrated an optimal Cmax/MIC ratio of 8–10, supporting its efficacy in treating MDR Gram-negative infections. Phase III studies are ongoing to confirm efficacy in complicated infections. Cefepime-based BL/BLI combinations are emerging as promising carbapenem-sparing agents, offering broad-spectrum activity, dual mechanisms of action, and encouraging clinical outcomes. These findings support their inclusion in antimicrobial stewardship strategies aimed at mitigating resistance.

## 1. Introduction

### 1.1. Epidemiological Context

The global spread of multidrug-resistant (MDR) Gram-negative bacteria represents a growing public health emergency, significantly complicating the treatment of severe infections and narrowing available antimicrobial options. In particular, extended-spectrum β-lactamase (ESBL)- and carbapenemase-producing *Enterobacterales*, *Pseudomonas aeruginosa*, and *Acinetobacter baumannii* are associated with increased morbidity, mortality, prolonged hospital stays, higher healthcare costs, and frequent therapeutic failures [1,2]. These pathogens are major causes of hospital- and healthcare-associated infections, including pneumonia, bloodstream infections, and complicated urinary tract infections.

Cefepime, a fourth-generation cephalosporin, has historically played an important role in the management of serious Gram-negative infections due to its broad-spectrum activity and favorable pharmacokinetic/pharmacodynamic (PK/PD) profile [3,4]. Its clinical utility has supported its use as a carbapenem-sparing option in antimicrobial stewardship programs [1,5]. However, the increasing global prevalence of ESBL- and carbapenemase-producing organisms has progressively limited the effectiveness of cefepime monotherapy, creating an urgent need for novel therapeutic strategies [2,6].

### 1.2. Mechanisms of Resistance and Therapeutic Innovations

Resistance to β-lactam antibiotics among Gram-negative pathogens is primarily driven by β-lactamase production, efflux pump overexpression, and alterations in outer-membrane porins, all of which have undermined the activity of traditional β-lactams, including cefepime [7,8]. Although cefepime demonstrates enhanced stability against AmpC β-lactamases and improved penetration through the outer membrane, these advantages are insufficient in the presence of ESBLs and carbapenemases [2,6]. To overcome these resistance mechanisms, novel β-lactam/β-lactamase inhibitor (BL/BLI) combinations have been designed to restore and enhance cefepime activity. Cefepime/enmetazobactam pairs cefepime with a penicillanic acid sulfone β-lactamase inhibitor that effectively inhibits class A ESBLs and improves antibacterial potency through its zwitterionic structure [2].

Cefepime/taniborbactam and cefepime/zidebactam represent further advances against MDR *Enterobacterales* and *Pseuodomonas aeruginosa*. Taniborbactam, a bicyclic boronate inhibitor, exhibits broad activity against serine β-lactamases (Ambler classes A, C, and D) and selected metallo-β-lactamases (MBLs), including NDM and VIM [2]. Zidebactam, a diazabicyclooctane (DBO) derivative, functions both as a β-lactamase inhibitor and as a high-affinity binder of penicillin-binding protein 2 (PBP2), resulting in synergistic bactericidal activity when combined with cefepime [2]. Cefepime/nacubactam combines cefepime with a second-generation DBO β-lactamase inhibitor that broadly inhibits class A and C, and some class D, enzymes while targeting PBP2. This dual mechanism provides intrinsic antibacterial activity and improves efficacy against difficult-to-treat non-fermenting Gram-negative pathogens, including *Pseudomonas aeruginosa* and *Acinetobacter baumannii* [2].

Preclinical studies indicate that cefepime/nacubactam has favorable PK/PD properties and potent in vivo activity, with phase III trials ongoing to assess its safety and efficacy in complicated MDR Gram-negative infections [2]. These results highlight the role of cefepime-based BL/BLI combinations in countering resistance and supporting carbapenem-sparing strategies in antimicrobial stewardship.

## 2. A Global Overview of Genes Encoding Resistance to Anti-Gram-Negative β-Lactams Antibiotics

Antimicrobial resistance among Gram-negative bacteria has a profound impact on multiple clinical outcomes, particularly in critically ill patients. While mortality remains the most alarming endpoint, resistance also significantly affects the length of hospital stay, healthcare costs, recurrence rates, and microbiological clearance [9]. Importantly, the increased mortality associated with MDR pathogens is often not due to greater intrinsic virulence, but rather to delays in initiating appropriate targeted therapy and the frequent need to rely on suboptimal antimicrobial regimens. In a large cohort study of 237 intensive care unit (ICU) patients, the overall ICU mortality rate significantly increased in patients with MDR infections. *Acinetobacter baumannii* was associated with the highest risk of death (odds ratio [OR] 4.6, *p* < 0.001), followed by methicillin-resistant *Staphylococcus aureus* (MRSA) (OR 3.5, *p* = 0.005), *Pseudomonas aeruginosa* (OR 2.8), and *Klebsiella pneumoniae* (OR 2.2). Among resistance mechanisms, carbapenemase-producing *Enterobacterales* showed the strongest association with mortality (OR 3.9), followed by AmpC-producing (OR 2.4) and ESBL-producing strains (OR 1.8) [10]. Complementing these findings, in another large retrospective cohort study of 1049 patients with BSIs, Santoro et al. demonstrated that extensively drug-resistant (XDR) Gram-negative bacteria significantly impacted outcomes at all time points. Specifically, XDR *Pseudomonas aeruginosa* was associated with a 7-day hazard ratio (HR) of 6.72. XDR *Acinetobacter baumannii* and carbapenem-resistant *Klebsiella pneumoniae* were also associated with elevated 30- and 90-day mortality [11].

## 3. Cefepime

### 3.1. Chemical Structure and Mechanism of Action

**Figure 1:**
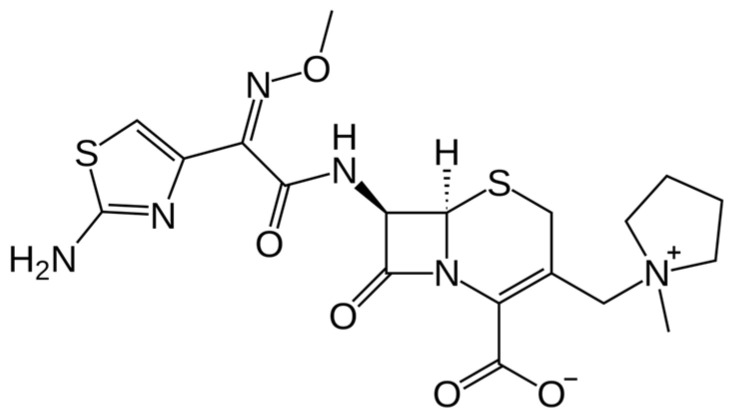
Chemical structure of cefepime.

Cefepime has been employed in the treatment of severe bacterial infections for nearly three decades. The emergence of ESBLs has posed a challenge to its continued use, although future co-administration with BLIs in development is an area of active research. Cefepime is a zwitterionic oxymino β-lactam with an amino-thiazole side chain, belonging to the fourth-generation cephalosporins. It is a compound, bearing (1-methylpyrrolidinium-1-y1-methyl and (2Z)-2-(2-amino-1,3-thiazol-4-yl)-2-(methoxyimino) acetamido groups at positions 3 and 7, respectively, of the cephem skeleton (Figure 1) [4].

**Scheme 1:**
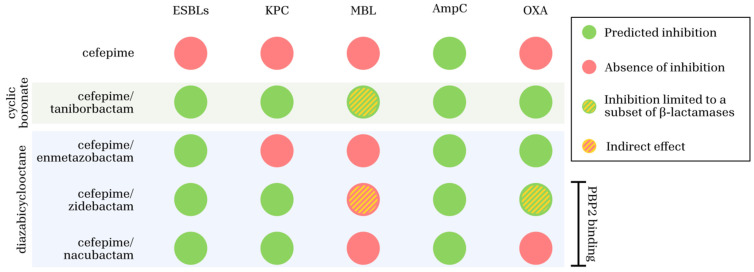
Cefepime and cefepime/inhibitor target activity.

Cefepime has broad-spectrum bactericidal activity against both Gram-positive and Gram-negative pathogens (Table 1, Scheme 1). Its activity against Gram-positive bacteria includes streptococci, such as *Streptococcus pneumoniae* and methicillin-susceptible *Staphylococcus aureus* (MSSA), whereas it is not active against MRSA or *Enterococcus* spp. [2]. Cefepime is highly effective against a variety of Gram-negatives, including *Pseudomonas aeruginosa* and *Enterobacterales* such as *Escherichia coli* and *Klebsiella pneumoniae*. The broader spectrum of activity compared to other generations of cephalosporins is due to its methylpyrrolidinium group that imparts a zwitterionic charge, which enhances rapid outer-membrane penetration, through the porin channels. Moreover, cefepime shows higher stability against different beta-lactamases, including AmpC (both chromosomal and plasmid-mediated), several ESBLs and oxacillinase (OXA)-48-like enzymes [1,3,7].

### 3.2. Microbiological Target

Cefepime inhibits bacterial cell-wall biosynthesis by covalently binding to penicillin-binding proteins (PBPs), thereby hindering the final transpeptidation stage of peptidoglycan synthesis. It primarily targets penicillin-binding proteins (PBPs) 2 and 3 in *Staphylococcus aureus*; however, its low affinity for PBP2a in MRSA (*methicillin-resistant Staphylococcus aureus*) strains contributes to resistance. Conversely, it has limited activity against *Enterococcus* spp. owing to its low PBP affinity. In Gram-negative pathogens, the efficacy of cefepime is related to the binding to PBP3, while additional affinity for PBP2 is described for *Enterobacterales* [2]. The strong activity of cefepime against strains producing AmpC β-lactamases, combined with its low potential to induce resistance mechanisms, makes it a valuable option for the treatment of infections caused by pathogens with chromosomal AmpC, such as *Pseudomonas aeruginosa* and *Enterobacter* spp. Among the most prevalent ESBLs, a subset of CTX-M enzymes, including, for example, CTX-M 14 [6], shows a lower affinity for cefepime, and organisms producing these ESBLs may appear susceptible in vitro, with MIC values lower than 8 mg/L. On the other hand, clinical efficacy is uncertain, and use of cefepime in such cases is controversial, suggesting that this drug has to be considered as a suboptimal option against ESBL-producing *Enterobacterales*, and MIC values should always be interpreted within the clinical context [3]. The activity of cefepime against anaerobes is limited, and therefore it is not recommended for infections involving obligate anaerobic bacteria without combination therapy [5]. Alterations in PBPs of Gram-positive bacteria represent the primary mechanism conferring resistance to cefepime. Cefepime exhibits low affinity for PBP2a in MRSA strains and for PBP4/PBP5 in *Enterococcus faecalis* and *Enterococcus faecium*, respectively [8,12]. Cefepime resistance among *Enterobacterales* predominantly arises through the production of β-lactamases. Cefepime exhibits stability against chromosomal or plasmid-mediated AmpC-type TEM- SHV CTX-M β-lactamases, as well as carbapenemases, excluding OXA-48 [13]. Furthermore, cefepime undergoes hydrolysis at a slower rate than ceftazidime by carbapenemases belonging to both Ambler class A and class B. The prevalence of CTX-M-type ESBLs has contributed to the increase in cefepime resistance [14]. In *Pseudomonas aeruginosa*, resistance to cefepime is due to chromosomal AmpC hyperproduction, stable derepression, and/or upregulation of efflux pumps; however, full resistance is associated with higher levels of AmpC β-lactamases [15]. A prevalent phenotype observed in *Pseudomons aeruginosa* is resistance to cefepime and susceptibility to ceftazidime, ascribed to the overexpression of the MexXY-OprM efflux system [16]. In *Acinetobacter baumannii*, cefepime resistance is due to a combination of chromosomal OXA-51/69-like carbapenemase hyperproduction, activation of efflux pumps (AdeABC) and probable porin changes [17]. The expression of OXA-type carbapenemases alone does not result in high-level cefepime resistance [18].

Cefepime has a broad microbiological spectrum, especially against Gram-negative pathogens, and its stability against many β-lactamases, mainly AmpC, and enhanced permeability make it a valuable choice for empiric and targeted treatment of different serious infections, not only alone but also as a part of a carbapenem-sparing strategy.

### 3.3. Clinical Use

Cefepime is an old fourth-generation anti-pseudomonal cephalosporin that has been available since the late 1990s, with extensive clinical experience supporting its use [1]. While its most common use remains in the treatment of pneumonia, both community- and hospital-acquired, cefepime is also frequently employed in cases of bacteremia and central nervous system infections, particularly following neurosurgical procedures. One of the key advantages of cefepime over earlier agents such as ceftazidime is its reliable activity against AmpC-producing Gram-negatives [19]. Its ability to maintain low MICs in this setting makes it particularly valuable in the empirical management of febrile neutropenia, where both *Pseudomonas* and AmpC producers are concerns [20]. Cefepime has shown a reliable safety profile over decades of use. Nonetheless, some caution is warranted in patients with impaired renal function, particularly given its moderate central nervous system penetration. Cases of neurotoxicity have been reported, most often in elderly patients with renal insufficiency and high plasma drug levels [21]. From a pharmacological perspective, cefepime remains stable at room temperature for 24 h, allowing for continuous infusion strategies that optimize its pharmacodynamic target [22]. These features also make it a suitable choice for outpatient parenteral antimicrobial therapy, especially in cases such as *Pseudomonas aeruginosa* osteomyelitis when fluoroquinolones are not an option due to resistance or intolerance [22]. Finally, cefepime has minimal activity against anaerobic flora, which also translates into a lower ecological impact on gut flora and a reduced risk of *Clostridioides difficile* infection, a non-negligible benefit in the current era of antimicrobial stewardship [23].

### 3.4. PK/PD Characteristics

The clinical PK and PD of cefepime have been recently reported [24]. Absorption is not a concern, as the drug is administered either as a short-term (30–60 min) or continuous/prolonged intravenous infusion (3 to 24 h). Cefepime is widely distributed in biological fluids and tissues. Compared to plasma, tissue penetration in the lungs, abdomen, joints, and bones reaches approximately 60–100%. Conversely, the cerebrospinal fluid (CSF)/plasma ratio is below 30%. The volume of distribution (Vd) is approximately 20 L in healthy adults with normal renal function. Protein binding is relatively low (20%), and elimination is predominantly renal. About 85% of the administered dose is excreted unchanged in the urine, with a half-life of 2–2.3 h. Cefepime is significantly removed by hemodialysis, and its clearance is directly proportional to dialysis flow rate and filter efficiency. During continuous renal replacement therapy, CL often approximates that of patients with normal kidney function, necessitating more frequent dosing [24]. Growing evidence shows that cefepime PK is altered under various pathophysiological conditions, such as impaired renal function, augmented renal clearance, dialysis, fluid shifts, pneumonia, febrile neutropenia, and in burn patients, critically ill or elderly individuals [24,25,26,27]. These findings present challenges for population-based dosing strategies, potentially increasing the risk of drug toxicity or reduced efficacy. Therefore, therapeutic drug monitoring (TDM) of cefepime plasma concentrations has proven valuable, especially in patients at high risk of cefepime-induced neurotoxicity, or those who are critically ill, have life-threatening infections, or are infected with resistant pathogens [28,29,30,31]. Over the past decade, several studies have aimed to correlate cefepime systemic exposure with the onset of neurotoxicity [28,29]. These studies consistently found that the risk of neurotoxicity increases significantly when trough plasma concentrations exceed 35–45 mg/L. However, these data largely concern cefepime administered via intermittent intravenous infusion. Increasingly, cefepime is being used as continuous or prolonged infusion in the treatment of severe infections or in complex patients. With this mode of administration, average plasma concentrations should not exceed 60 mg/L to reduce the risk of cefepime-related neurotoxicity [28,29]. As a member of the beta-lactam (BL) class of antibiotics, cefepime is considered a time-dependent agent, with antibacterial activity relying on the duration that free drug concentrations remain above the minimum inhibitory concentration (fT > MIC). However, there is considerable variability in reported efficacy targets [24]. Recent evidence suggests that optimal PK/PD indices for microbiological and clinical success may be higher than the previously established preclinical target of 40–70% fT > MIC, although a consensus is currently lacking. Specifically, a target of 75–100% fT > MIC has been proposed as a favorable predictor of treatment outcomes in critically ill adults, patients with sepsis, and the elderly. Moreover, more aggressive PK/PD targets (e.g., 100% fT > 4–8 × MIC) have been advocated to prevent resistance development [32]. Based on the current literature, it is proposed that the free cefepime trough or average concentrations be maintained above the MIC for 100% of the dosing interval, with more aggressive targets (i.e., 100% fT > 5 × MIC) considered in severe clinical scenarios.

## 4. Cefepime/Enmetazobactam

### 4.1. Chemical Structure and Mechanism of Action

Cefepime/enmetazobactam is a novel beta-lactamase inhibitor combination of a 4th generation cephalosporin and an ESBL inhibitor for MDR Gram-negative bacteria [33]. *Enterobacterales* EUCAST 2024 guidelines are fixed for cefepime/enmetazobactam combination (4–4 mg/L, 22–22 mm).

According to ISO 20776–1 [34], MIC determination by broth microdilution can be achieved in unsupplemented cation-adjusted Mueller–Hinton broth with an established concentration of 8 mg/L of enmetazobactam.

### 4.2. Microbiological Target

**Figure 2:**
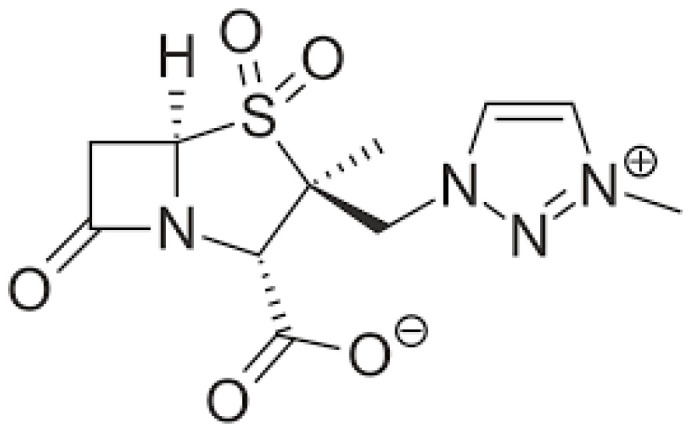
Chemical structure of enmetazobactam.

Cefepime, as previously reported, exhibits broad activity against Gram-positive and Gram-negative pathogens (including *Pseudomonas* species) and AmpC-producing *Enterobacterales* for its affinity to penicillin-binding proteins (PBP2 and PBP3), with the exception of enterococci and anaerobes. ESBL enzymes other than AmpC β-lactamase are able to hydrolyze cefepime and are frequently associated with clinical failure [35,36,37]. The addition of enmetazobactam extends cefepime’s spectrum of activity further to include ESBL-producing *Enterobacterales*. Enmetazobactam (formerly AAI101, Figure 2) is a penicillanic acid sulfone-based beta-lactamase inhibitor that protects cefepime from degradation by certain SBLs. The key difference of enmetazobactam with respect to tazobactam is its improved potency due to a methyl group on the triazole ring that imparts a net neutral charge (zwitterionic), allowing for many classical and non-classical hydrogen bonding interactions and enhancing bacterial cell penetration [38]. In *Enterobacterales*, cefepime/enmetazobactam exhibits activity against CTX-M, TEM, SHV, and VEB beta-lactamases, while it does not act against KPC, MBL, or some OXA [39]. Some limitations in the spectrum of activity include *Stenotrophomonas maltophilia*, MDR *Pseudomonas* species, MRSA, *Enterococcus* spp., and anaerobes. In isolates of *Acinetobacter baumannii*, both wild-type and carbapenem-non-susceptible, enmetazobactam did not demonstrate antimicrobial activity. [40]. Mechanisms of resistance to cefepime/enmetazobactam involve production of metallo-β-lactamases, reduced intracellular drug concentration (porin mutations and efflux pump overexpression) and mutations in the PBP genes [41].

### 4.3. Clinical Use

Cefepime/enmetazobactam is a BL/BLI combination with potent activity against ESBL-producing *Enterobacterales*. The US FDA has recently approved it for the treatment of cUTIs. Meanwhile, the European Medicines Agency (EMA) and the UK Medicines and Healthcare products Regulatory Agency (MHRA) have extended the authorization of cefepime/enmetazobactam also to include hospital-acquired pneumonia (HAP) [including ventilator-associated pneumonia (VAP)] and bacteremia in adults [41]. Two phase I studies (NCT03680352 and NCT03775668), one phase II study (NCT03680612), and one phase III multi-center randomized controlled trial (ALLIUM) have been completed [42]. The phase III ALLIUM trial compared 2 g cefepime plus 500 mg enmetazobactam (with 2 h of infusion) every 8 hours with piperacillin/tazobactam 4.5 g (with 2 h of infusion) every 8 h for 7 days for the treatment of cUTIs, demonstrating the superiority of cefepime/enmetazobactam in the primary composite outcome, defined as clinical cure (resolution of baseline signs and symptoms) and microbiological cure (reduction in baseline urinary pathogen concentration to <10^3^ CFU/mL by day 14). The difference between the two groups was 79.1% vs. 58.9%. Although clinical cure rates alone were similar between groups, cefepime/enmetazobactam achieved significantly higher microbiological eradication rates (82.9% vs. 64.9%), particularly in infections caused by *Enterobacterales* ESBL (73.7% vs. 51.5%). Microbial recurrence was substantially lower in the cefepime/enmetazobactam group (11.35% vs. 29.4%), with no significant differences in adverse events between the treatment arms [43]. Although no dedicated phase III clinical studies are currently available to support the use of cefepime/enmetazobactam in pneumonia, approval by the EMA and MHRA is based on PK/PD extrapolation, supported by preclinical evidence from murine models indicating a favorable pharmacokinetic/pharmacodynamic profile in the pulmonary setting, including adequate penetration into lung tissue and epithelial lining fluid (ELF) [41,44]. The recommended dose for patients with normal renal function in cases of HAP and VAP is 2 g/0.5 g administered every 8 h as an intravenous infusion over 4 h. For cUTIs, including pyelonephritis, the same dosage (2 g/0.5 g every 8 h) is recommended, but administered as an intravenous infusion over 2 h [45].

### 4.4. PK/PD Characteristics

In this section, we focus on enmetazobactam, as the PK and PD of cefepime were discussed in the previous chapter. Enmetazobactam distributes widely throughout body compartments, with a volume of distribution of approximately 21 L and negligible protein binding (Table 2). A study conducted in healthy volunteers reported a ratio of epithelial lining fluid (ELF) to plasma concentration of 53% for enmetazobactam [46]. Currently, no data are available regarding its distribution in CSF, bone, or soft tissues. Enmetazobactam is primarily excreted renally, with 90% of the administered dose recovered unchanged in urine over a 24 h period, and it undergoes negligible hepatic metabolism [41,42]. Dose adjustments are recommended for patients with renal impairment. Specifically, for patients with an estimated glomerular filtration rate (eGFR) of 30–59 mL/min, a reduced dosing regimen of 1 g/0.25 g every 8 h is advised; for those with an eGFR of 15–29 mL/min, 1 g/0.25 g every 12 h; and for those with an eGFR < 15 mL/min, 1 g/0.25 g every 24 h. In patients with augmented renal clearance (eGFR > 150 mL/min), systemic drug exposure was reduced by approximately 28%. Therefore, in these cases, extending the infusion duration to 4 h (instead of the standard 2 h) is recommended. For patients undergoing hemodialysis, a loading dose of 1 g/0.25 g is administered every 24 h, followed by 0.5 g/0.125 g every 24 h thereafter, with administration post-dialysis on dialysis days. No dosage recommendations are currently available for patients receiving continuous renal replacement therapy. In those undergoing continuous ambulatory peritoneal dialysis, the recommended parenteral regimen is 2 g/0.5 g every 48 h. No dose adjustment is required in patients with hepatic impairment or in the elderly. Monte Carlo simulations based on a population PK model derived from pooled data from phase 1–3 clinical studies demonstrated that approved cefepime/enmetazobactam regimens achieve a probability of target attainment >90% against a broad MIC distribution (up to 8 mg/L) of *Enterobacterales* [42]. Additionally, these simulations indicated that achieving enhanced PD targets in ELF—specifically, 60% of the dosing interval with fT > MIC and 45% of the interval with free enmetazobactam concentrations above 2 mg/L—requires a 4 h infusion in patients with pneumonia, as defined by the European drug label [42]. To date, no therapeutic drug monitoring (TDM) studies assessing plasma concentrations of enmetazobactam have been published.

## 5. Cefepime/Taniborbactam

### 5.1. Chemical Structure and Mechanism of Action

**Figure 3:**
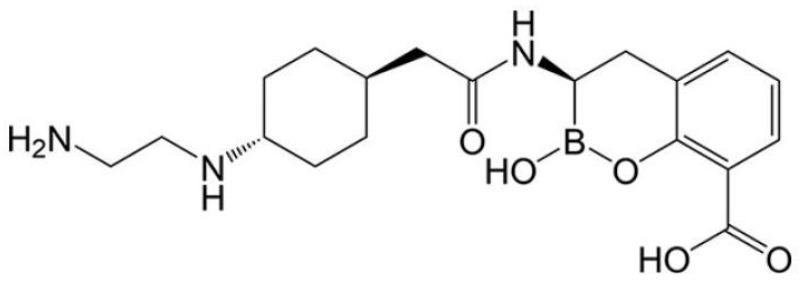
Chemical structure of taniborbactam.

Cefepime/taniborbactam combination is a 4th generation cephalosporin combined with the bicyclic boronate VNRX-5133 (Figure 3) [47]. The boron atom present in the structure of taniborbactam acts as potent inhibitor of both SBLs and MBLs. This property is due to the ability of the boron element to rapidly switch between different hybridization states during catalysis [48]. The boronate can mimic the acylation or diacylation states of the reactions mediated by SBLs and MBLs, depending on β-lactam hydrolysis. Kinetic data for VNRX-5133 are available only for selected SBLs and MBLs.

However, MIC values determined against class A, B, C and D β-lactamases showed that taniborbactam is able to restore the activity of cefepime in more than 90% of β-lactamase-producing isogenic *Escherichia coli*, as well as in a high percentage of β-lactamases produced by *Enterobacterales* and *Pseudomonas aeruginosa* [49,50]. In this review, we discuss the kinetic profiles of selected SBLs, including class A (KPC-variants, CTX-M-15, SHV-5), class D (e.g., OXA-48), and class C (e.g., AmpC) enzymes, as well as MBLs such as NDM-1 and its variants, VIM-2 and IMP-1, for which data are currently available. Concerning class A BLs, taniborbactam efficiently inhibits KPC-2 with an inhibition constant in the nanomolar range (*K_i_* = 4 nM) [50]. Meyers et al. reported the inhibition constants of taniborbactam against KPC-2, KPC-3 and KPC-variants V240G, D179Y and D179Y/T243M and compared them to those of ledaborbactam and avibactam (AVI) [51]. In detail, taniborbactam inactivates KPC-2 and KPC-3 with inactivation efficiencies (*k*_2_/*K*) of 0.9 × 10^4^ M^−1^s^−1^ and 4.0 × 10^4^ M^−1^s^−1^, respectively [51]. The behavior of KPC-2/KPC-3 variants differs slightly from the wild-type variant. In fact, the inactivation efficiencies of taniborbactam against V240G, D179Y and D179Y/T243M variants are reduced [51]. Meyers et al. reported molecular modeling experiments showing that the reduced efficiency of taniborbactam in these variants is due to the loss of key hydrophobic interactions with tryptophan at position 105 (W105) [51]. The extended-spectrum CTX-M-15 and SHV-5 β-lactamases are efficiently inhibited by taniborbactam with *K_i_* values of 17 nM and 3 nM, respectively [44]. Liu and coauthors demonstrated that the boron atom of taniborbactam covalently binds the Ser70 of CTX-M-15, adopting a tetrahedral conformation [48]. The chromosomal class C enzymes are also inhibited by taniborbactam; an example is represented by P99AmpC, which showed a *K_i_* of 2 nM [50]. Lang et al. reported *K_iapp_* and *k*_2_/*K* values for taniborbactam against AmpC of *Escherichia coli* of 3.72 µM and 85.6 × 10^3^ M^−1^ s^−1^, respectively [52]. The crystal structure of AmpC of *Escherichia coli* complexed with taniborbactam showed that the bicyclic boronate reacts with the nucleophilic active site Ser64 [52]. Regarding class D and B carbapenemases, taniborbactam is a potent inhibitor of OXA-48 (*K_i_* = 350 nM), NDM-1 (*K_i_* = 81 nM) and VIM-2 (*K_i_* = 19 nM) [50]. In NDM-1 enzymes, the loop L3 is involved in binding with substrates and/or inhibitors, and when NDM-1 is co-crystalized with taniborbactam, the L3 is partially disordered, and it is unable to stabilize the complex [47]. Taniborbactam efficiently inhibits more NDM-1 variants [50,53]. However, some NDM-1 variants such as NDM-1^K224I^ showed a decrease in the catalytic efficiency against cefepime and an increase in *K_i_* for taniborbactam with respect to wild-type NDM-1 [54]. Some MBLs such as IMP-1, NDM-9, NDM-30 and VIM-83 have been found to be resistant to taniborbactam [55,56].

### 5.2. Microbiological Target

Taniborbactam, also known as VNRX-5133, is a β-lactamase inhibitor of the cyclic boronate class. It inhibits serine β-lactamases (SBLs; classes A, C, and D) and selected class B1 metallo-β-lactamases (MBLs) such as VIM, NDM, SPM-1, and GIM-1, although activity may be limited against certain NDM variants and is minimal or absent against IMP and SIM-like enzymes, as well as class B2/B3 MBLs [57]. Combined with cefepime, it forms a broad-spectrum antimicrobial (FTB) active against carbapenem-resistant *Enterobacterales* and *Pseudomonas aeruginosa*, including MDR and difficult-to-treat resistant (DTR) strains. FTB retains activity against isolates resistant to other BL/BLI combinations, such as ceftolozane/tazobactam, ceftazidime/avibactam, and meropenem/vaborbactam, and has demonstrated efficacy in murine models of cUTIs and pneumonia, including strains non-susceptible to cefepime alone [57].

Surveillance studies (e.g., GEARS) and in vitro testing of over 28,000 clinical isolates show high susceptibility rates, though structural variations in certain NDM variants can reduce activity by impairing enzyme inhibition. Clinically, this may result in lower efficacy against infections caused by these variants compared with strains harboring more common NDM types, such as NDM-1. MIC values are generally more favorable in VIM-positive than in NDM-positive isolates, with notable geographic variability.

Resistance can arise via MBL mutations affecting the active site or through non-enzymatic mechanisms, including PBP-3 mutations, porin loss, or efflux pump overexpression. Kinetic studies show that taniborbactam competitively inhibits enzymes such as VIM-2 and NDM-1 with high affinity, while activity against IMP-type enzymes remains limited [58]. FTB is inactive against *Acinetobacter baumannii* but shows efficacy against *Stenotrophomonas maltophilia*—due to cefepime stability and L2 β-lactamase inhibition—and exhibits in vitro activity against the *Burkholderia cepacia complex*.

Overall, taniborbactam represents a significant advancement against multidrug-resistant Gram-negative pathogens, although continuous monitoring of emerging resistance mechanisms is essential to maintain its clinical utility [59].

### 5.3. Clinical Use

Taniborbactam is a novel bicyclic boronate β-lactamase inhibitor with activity against Ambler classes A, B, C, and D, with a potent effect against MBLs, such as VIM and NDM [60]. The combination of cefepime and taniborbactam demonstrates in vitro activity against most carbapenem-resistant *Enterobacterales* (CRE) and carbapenem-resistant *Pseudomonas aeruginosa* (CRPA) isolates, encompassing both carbapenemase-producing and non-producing strains. It is also active against MDR *Enterobacterales* and *Pseudomonas aeruginosa* resistant to ceftazidime/avibactam, meropenem/vaborbactam, and ceftolozane/tazobactam [61]. The clinical efficacy of cefepime/taniborbactam was demonstrated in a phase III, double-blind, randomized trial (CERTAIN-1). Patients were randomly assigned in a 2:1 ratio to receive either cefepime/taniborbactam 2.5 g administered over a 2 h infusion every 8 h, plus a meropenem placebo, or meropenem at a dose of 1 g administered over 30 min every 8 h, plus a cefepime–taniborbactam placebo, for 7 days. For patients with bacteremia, the treatment duration could be extended up to 14 days [56]. The study demonstrates the superiority of cefepime/taniborbactam regarding composite (both microbiologic and clinical) success at the test of cure among hospitalized adults undergoing treatment for cUTI, including acute pyelonephritis caused by *Enterobacterales* and *Pseudomonas aeruginosa* [58,62]. Overall, cefepime/taniborbactam appears to be well tolerated, with a safety profile similar to that of meropenem [58]. Moreover, headache and gastrointestinal disturbances were reported as the most common treatment-related adverse effects [58,61]. Additional studies have been planned, including CERTAIN-2, a phase 3 randomized controlled trial designed to compare cefepime/taniborbactam with meropenem in patients with ventilator-associated bacterial pneumonia. However, this trial is currently suspended [63].

### 5.4. PK/PD Characteristics

The first-in-human study evaluating the PK of single (62.5 to 1500 mg) and multiple doses (250 to 750 mg every 8 h for 10 days) of taniborbactam in healthy adult subjects was published by Dowell and coauthors [64]. All doses of taniborbactam were administered as a 2 h intravenous infusions. Taniborbactam exhibited dose-proportional PK with low intersubject variability (main PK parameters are reported in Table 3). The same group subsequently assessed the PK of taniborbactam in subjects with varying degrees of renal impairment [65]. Compared to subjects with normal renal function, mean taniborbactam clearance decreased by 15%, 63%, and 81% in subjects with mild, moderate, and severe renal impairment, respectively. Taniborbactam was also found to be dialyzable. Therefore, dose adjustments are necessary based on the degree of renal impairment. Asempa and coauthors evaluated the bronchopulmonary disposition of intravenous cefepime/taniborbactam (2 g/0.5 g) administered over 2 h in healthy adult subjects [66]. Mean taniborbactam AUC in plasma, ELF, and alveolar macrophages (AM) were 81.9, 13.8, and 77.9 mg·h/L, respectively, corresponding to ELF/plasma and AM/plasma ratios of 17.9% and 95.1%. These findings were partially confirmed by Rodvold and coauthors, who reported ELF/plasma ratios for taniborbactam ranging from 15.3 to 25.3% [67]. Using an in vitro PK model, Noel et al. recently demonstrated that the primary PD drivers of taniborbactam are the AUC and the %fT > MIC, both closely linked to antibacterial activity [68]. Specifically, the taniborbactam AUC required to achieve a 1-log_10_ reduction in viable bacterial count ranged from 4.4 to 11.2 mg·h/L. To date, PK/PD studies in humans are lacking.

## 6. Cefepime/Zidebactam

### 6.1. Chemical Structure and Mechanism of Action

**Figure 4:**
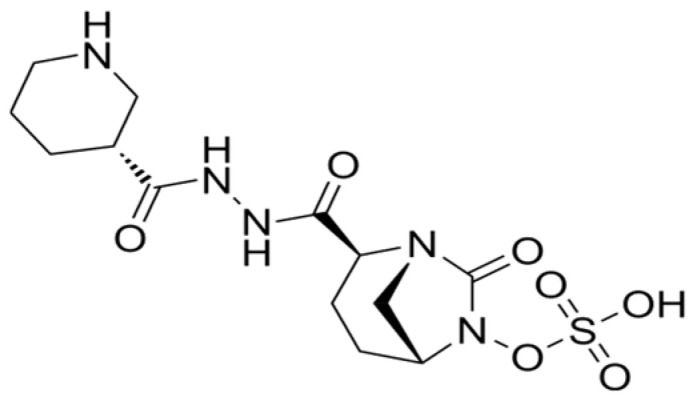
Chemical structure of zidebactam.

At the beginning of 2000s, a new class of BLIs, the DBO compounds, was introduced into clinical practice [69]. Important examples of this class of BLIs are avibactam and relebactam which can inhibit some metallo-β-lactamases, KPC-carbapenemases, and class D as well as class C enzymes [70,71]. Zidebactam (Figure 4) and nacubactam are the “second generation” of DBOs, which enhanced activity against both β-lactamases and PBPs [72]. Cefepime/zidebactam is a 4th-generation cephalosporin combined with a DBO β-lactamase inhibitor. This combination has been found to inhibit bacterial cell-wall synthesis by binding to PBP2 as well as inhibiting certain β-lactamases [57]. In Gram-negative bacteria, zidebactam induces spheroplast formation and, when used in combination with cefepime, leads to cell lysis [73]. To date, the kinetic profile of zidebactam against selected β-lactamases is not available. However, it has been proven that zidebactam acts as potent inhibitor against class A and C β-lactamases produced by *Enterobacterales* and *Pseudomonas aeruginosa* [73,74].

Kinetic data for zidebactam against OXA-48 showed a *K_iapp_* > 800 μM, comparable to other DBOs such as nacubactam and relebactam [75]. Moreover, zidebactam exhibited weaker binding affinity for OXA-48 as indicated by a high dissociation constant (*K_d_* = 1.37 × 10^3^) and a low association rate (*k*_2_/*K* = 20.11) [75]. Interactions between zidebactam and PBPs were characterized, and *IC*_50_ values were determined for PBP1a, PBP1b, PBP2, PBP3, PBP4 and PBP5/6 [73]. Notably, the *IC*_50_ for PBP2 (0.26 µg/mL) was significantly lower than those determined for the other PBPs. The structural analysis of *Pseudomonas aeruginosa* PBP-2 revealed that this protein has four distinct regions protruding into the periplasm, with its transpeptidase domain cross-linking the nascent peptidoglycan strand [76]. Crystal structure studies showed that DBOs interact with active site motifs 327S-X-X-K330, 384S-X-N/D386 and 538K-T/S-G-T541of PBPs [76]. Specifically, S327 of PBP2 covalently binds the carbonyl oxygen of zidebactam, leading to the formation of the acyl-enzyme inhibited state [77]. Similarly, the carbonyl oxygen of zidebactam, located in the PBP oxyanion hole, is covalently bound to the active serine S294 of PBP3 [76]. Despite the differences between the active site motifs of PBP2 and PBP3, the diacilhydrazide moiety of DBO interacts with the S-X-D/N motif. In addition, the oxygen atom of zidebactam interacts via hydrogen bond with a water molecule [76].

### 6.2. Microbiological Target

Zidebactam is a non-β-lactam antibiotic with a microbiological mechanism involving selective and high-affinity Gram-negative PBP2 binding and β-lactamase inhibition, and it is not hydrolysed by β-lactamases, including MBLs and class D enzymes. It has been designed to increase PBP2 binding in Gram-negative species, especially *Pseudomonas aeruginosa* and *Acinetobacater baumannii*. Its high affinity for PBP2 promotes the inhactivation of the peptidoglycan layer formation process, enhancing the antimicrobial action of other beta-lactams in combination. Synergistic activity is obtained with zidebactam in combination with a PBP3-targeting β-lactam, such as cefepime. This combination showed antimicrobial activity against isolates producing ESBL and AmpC variants, KPC enzymes, class D OXA β-lactamases and/or MBLs [73,78,79]. Zidebactam with cefepime in a 1:1 combination is in clinical development for the treatment of Gram-negative bacterial infections. Literature data showed that cefepime/zidebactam was highly active against *Enterobacterales* (MIC_50/90_ 0.03/0.25 mg/L) and *Pseudomonas aeruginosa* (MIC_50/90_ 1/4 mg/L) (Table 2). In these species, cefepime/zidebactam showed higher antimicrobial activity than meropenem, ceftazidime/avibactam and amikacin (*Enterobacterales*) and ceftazidime/avibactam, ceftolozane/tazobactam and tobramycin (*Pseudomonas aeruginosa*). In particular, in *Pseudomonas aeruginosa* it also retained activity against piperacillin/tazobactam-, ceftazidime/avibactam- and ceftolozane/tazobactam-non-susceptible isolates (MIC ≤ 8 mg/L) [80]. In the study of Thomson and coauthors, cefepime/zidebactam was highly active against *Pseudomonas aeruginosa* isolates with multiple resistance mechanisms, including combinations of upregulated efflux, diminished or non-functional OprD porins, and AmpC overproduction [81]. Lee and coauthors reported an important in vitro activity of cefepime/zidebactam against imipenem-non-susceptible *Pseudomonas aeruginosa* isolates, better than ceftazidime/avibactam, imipenem/relebactam and ceftolozane/tazobactam, respectively [82]. Petillon and coauthors reported an antimicrobial activity of cefepime/zidebactam similar to aztreonam/avibactam against ESBL- and carbapenemase-producing *Enterobacterales* [83]. Cefepime/zidebactam demonstrated potent activity with MIC50 and MIC90 of 1 and 2 mg/L, respectively, against a collection of *Klebsiella pneumoniae* isolates that produced OXA-48 and NDM carbapenemases [84]. Good activity has also been described against *Stenotrophomonas maltophilia* (MIC ≤ 8 mg/L) and *Burkholderia cepacia* complex. Relatively higher MIC_50/90_ values (16/32 mg/L) were reported for *Acinetobacter* spp. when compared with other Gram-negative organisms [80]. Elevated MIC values (>8 mg/L) were reported in *Escherichia coli* isolates that produced NDM-5 and carried a V522I substitution in PBP2 [85]. Additionally, Liu and coauthors reported elevated MIC values to cefepime/zidebactam (MIC > 8 mg/L) in *Klebsiella pneumoniae* isolates carrying NDM determinants and insertions in PBP3 or porin alterations [86]. Zidebactam alone showed no activity against *Chryseobacterium indologenes* and *Elizabethkingia meningoseptica* clinical isolates [87]. For antimicrobial susceptibility testing, zidebactam should be tested at a 1:1 concentration with cefepime [88]. No clinical breakpoint, as defined by CLSI, EUCAST, or FDA, has been established for this combination.

### 6.3. Clinical Use

Recent clinical investigations have explored the efficacy and safety of cefepime/zidebactam (WCK 5222), a novel β-lactam/β-lactam enhancer combination designed to target MDR Gram-negative pathogens. Three phase I studies (NCT02707107, NCT03630094, NCT03554304) were designed to evaluate the safety, tolerability, and pharmacokinetic profile of cefepime/zidebactam in healthy volunteers, including assessments of plasma and intrapulmonary concentrations; however, study results are not yet publicly available. Another phase I trial is currently ongoing in the United States (NCT06806995). This is a single-center, open-label, cross-over study designed to evaluate the safety and pharmacokinetics of single intravenous doses of cefepime/zidebactam (3 g), metronidazole (0.5 g), and their sequential administration in 30 healthy adult volunteers. To date, only one phase I clinical trial investigating cefepime/zidebactam has published results. In this single-center study (NCT02942810), the pharmacokinetics and safety of cefepime/zidebactam were assessed in 48 subjects with varying degrees of renal function, including end-stage renal disease requiring haemodialysis. Participants received either a 3 g dose (2 g cefepime + 1 g zidebactam) or a 1.5 g dose (1 g cefepime + 0.5 g zidebactam) via intravenous infusion, depending on renal status. The study demonstrated that drug clearance decreased and plasma exposure increased with worsening renal function, supporting the need for dose adjustment. Cefepime/zidebactam was well tolerated across all groups, with no significant safety concerns [89]. A phase III trial (NCT04979806) was recently completed, although results have not yet been made publicly available. The aim of the study was to evaluate cefepime/zidebactam in hospitalized adults with cUTI and acute pyelonephritis, assessing non-inferiority to meropenem in terms of clinical and microbiological outcomes. Furthermore, real-world evidence from compassionate use and case series, all published in India, has highlighted the potential of cefepime/zidebactam in treating extensively drug-resistant infections, including those caused by NDM-producing *Pseudomonas aeruginosa.* These case series collectively support the continued clinical development of cefepime/zidebactam as a promising agent against resistant Gram-negative pathogens [90,91,92,93,94,95].

### 6.4. PK/PD Characteristics

As shown in Table 3, zidebactam is characterized by a low Vd of 15–20 L, low level of plasma protein binding (<15%), and a short half-life, consistent with other BLIs [89]. Data on tissue penetration are currently available only for the respiratory tract: following multiple doses of cefepime 2 g plus zidebactam 1 g every 8 h, the ratio of ELF to total plasma penetration was 38%, while the ratio of alveolar macrophage to total plasma was 10% [96]. A single-center study evaluating the pharmacokinetics of cefepime/zidebactam was conducted by Preston et al., involving healthy controls and subjects with mild, moderate, and severe renal impairment, as well as patients on intermittent hemodialysis [89]. They observed that renal clearance decreased, while plasma t1/2 and AUC increased progressively with the severity of renal impairment for both drugs. Consequently, dosage adjustments are required in patients with renal failure. Lepak and coauthors evaluated the impact of zidebactam on the cefepime PK/PD target (%fT > MIC) required for efficacy against a diverse group of carbapenem-resistant *Enterobacteriaceae*-producing metallo-β-lactamases [97]. Dose-ranging studies were performed in lung-infected mice challenged with one of eight MBL-producing clinical strains. Cefepime/zidebactam was administered in regimens every 4 or 8 h to achieve %T > MIC exposures ranging from 0 to 100%. The data were modeled to evaluate the relationship between cefepime %T > MIC, in the presence of zidebactam, and therapeutic effect. Results revealed a strong correlation between %T > MIC and antibacterial effect. Net stasis in bacterial burden was achieved at cefepime %T > MIC exposures as low as 18%, while a 1-log_10_ kill endpoint was reached at approximately 31% %T > MIC. These target exposures for stasis and 1-log kill are substantially lower than those previously reported for cephalosporin monotherapy PK/PD targets.

## 7. Cefepime/Nacubactam

### 7.1. Chemical Structure and Mechanism of Action

**Figure 5:**
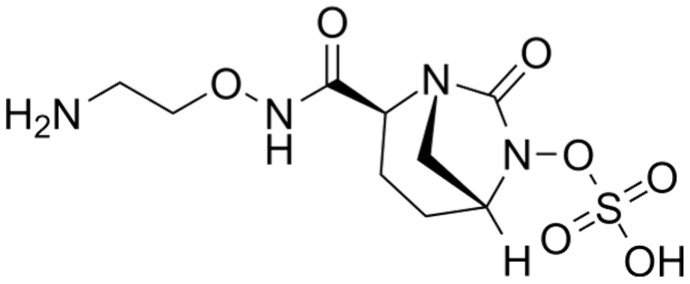
Chemical structure of nacubactam.

Nacubactam (RG6080/OP0595; Roche, Fedora, Meiji) (Figure 5) is a “second generation” of DBOs that acts both as a β-lactamase inhibitor and a binder of PBPs.

The inhibitory activity of nacubactam has been tested against various class A, C, B and D β-lactamases [98]. As reported by Morinaka and coauthors, nacubactam efficiently inhibits class A β-lactamases with *IC*_50_ values ranging from 9.49 nM to 869 nM. In detail, the TEM-1, TEM-10, CTX-M-14, CTX-M-15, CTX-M-44 and KPC-2 enzymes exhibited the *IC*_50_ values of 26.1 nM, 95.7 nM, 9.49 nM, 13.1 nM, 22 nM and 869 nM, respectively [98]. Nacubactam also inhibits CMY-2 (IC50 = 15 nM) more efficiently than the AmpC of *Escherichia coli* and *Pseudomonas aeruginosa*, which showed *IC*_50_s of 845 nM and 271 nM. In contrast, the OXA-23 (class D) and IMP-1 (class B) enzymes are highly resistant to inhibition by nacubactam. Notably, the *IC*_50_s determined for OXA-23 and IMP-1 were 46.4 μM and >300 μM, respectively [98]. Crystal structures of nacubactam in complexes with AmpC and CTX-M-44 are available [98]. In the AmpC/nacubactam complex, the carboxamide group of the inhibitor forms hydrogen bonds with the side chains of Q146 and N179, as well as with the carbonyl oxygen of S345. Similarly, in the CTX-M-44/nacubactam complex, the carboxamide group of nacubactam forms hydrogen bonds with the side chains of N104 and N132 and with a water molecule. A comparable interaction profile was observed in the CTX-M-15/nacubactam and AmpC/nacubactam complexes [99,100]. An important peculiarity of nacubactam was also observed: it acts as an “enhancer” of the activity of certain β-lactams that bind to PBPs, specifically PBP2 [98].

### 7.2. Microbiological Target

Cefepime/nacubactam demonstrated activity against a broad range of Gram-negative *microorganisms*, including beta-lactamase-producing *Enterobacterales* and difficult-to-treat non fermenting species such as *Acinetobacter baumannii* and *Pseudomonas aeruginosa* [101,102,103], effects observed not only in vitro but also in vivo [104]. Nacubactam plays a role not only as a beta-lactamase inhibitor, but also by binding to the PBP2 (hence displaying a bactericidal activity) and enhancing the activity of beta-lactams characterized by high affinity towards PBP3 [105]. Cefepime activity results greatly potentiated against *Enterobacterales* encoding different class A ESBLs but not against those carrying either chromosomal or plasmid-encoded AmpC beta-lactamases, plausibly owing to the scarce hydrolytic activity of AmpC towards cefepime [103]. At a fixed concentration of 4 mg/L, nacubactam also lowers the MIC for cefepime ≥ 8 fold in several *Enterobacterales* species (*Klebsiella pneumoniae*, *Morganella morganii*, *Enterobacter cloacae*, and *Escherichia coli*) producing Ambler class A (e.g., KPC), B (e.g., NDM) and class D (e.g., OXA-48) carbapenemases, even in isolates co-producing other ESBLs or AmpC enzymes, in most cases restoring full cefepime susceptibility [103,105]. A notable advantage of the cefepime/nacubactam combination against *Enterobacterales* is its effectiveness against strains with reduced outer-membrane permeability owing to alterations in non-selective porins, such as *Klebsiella pneumoniae* isolates with depleted OmpK35 or amino acid-mediated insertions leading to pore constriction in OmpK36 [101,103,105]. In a similar fashion, nacubactam fairly potentiates cefepime against *Pseudomonas aeruginosa* strains with derepressed AmpC but enhances its activity against those isolates with acquired PER or VEB beta-lactamases. However, this complementary effect is notably absent in class B metallo-β-lactamase (MBL)-producing *Pseudomonas aeruginosa*, reflecting the fundamental inability of nacubactam to inhibit zinc-dependent carbapenemases and its limited intrinsic activity against *Pseudomonas* targets [101,103,105]. In this context, the lack of direct MBL inhibition cannot be compensated by PBP binding or β-lactamase shielding, particularly in organisms that also exhibit reduced permeability and active efflux. Importantly, nacubactam confers little to no potentiation of cefepime against *Acinetobacter baumannii*, irrespective of the underlying β-lactam resistance mechanism, including chromosomally encoded class B and class D carbapenemases [103]. This consistent lack of activity against MBL-producing pathogens underscores a critical limitation of cefepime/nacubactam: while it represents a valuable therapeutic option against many MDR *Enterobacterales* and select *P. aeruginosa* isolates, it remains ineffective against MBL-driven resistance phenotypes. Consequently, infections caused by MBL-producing *P. aeruginosa* and *A. baumannii* continue to require alternative treatment strategies, highlighting a significant unmet need in the current β-lactam/β-lactamase inhibitor landscape.

### 7.3. Clinical Use

Recent clinical development efforts have focused on cefepime/nacubactam, a novel β-lactam/β-lactam designed to target MDR Gram-negative bacteria, including carbapenem-resistant *Enterobacterales*. There are currently no published case series or reports from real-world or compassionate-use settings involving cefepime/nacubactam. However, two phase III clinical trials have been initiated to investigate its efficacy and safety in patients with severe infections caused by carbapenem-resistant *Enterobacterales*. The Integral-1 trial (NCT05887908) was recently completed in Estonia; however, the results have not yet been published. The aim was to assess the efficacy and safety of cefepime/nacubactam or aztreonam/nacubactam compared to imipenem/cilastatin in the treatment of complicated urinary tract infections or acute uncomplicated pyelonephritis. The Integral-2 trial (NCT05905055) is currently ongoing. This multi-center, randomized, single-blind, parallel-group study is being conducted in Japan to evaluate the efficacy and safety of nacubactam co-administered with either cefepime or aztreonam, compared with best available therapy, in the treatment of patients with cUTI, acute pyelonephritis, hospital-acquired bacterial pneumonia, ventilator-associated bacterial pneumonia, and complicated intra-abdominal infections. These studies are expected to provide important insights into the potential role of cefepime/nacubactam in treating infections caused by MDR Gram-negative bacteria.

### 7.4. PK/PD Characteristics

The PK of intravenous nacubactam was evaluated in single- and multiple-ascending-dose, placebo-controlled studies [100]. Healthy participants received single ascending doses of nacubactam ranging from 50 to 8000 mg, multiple ascending doses of 1000 to 4000 mg every 8 h (q8h) for up to 7 days, or nacubactam 2000 mg q8h for 6 days following a 3-day lead-in period. Following both single and multiple doses, nacubactam exhibited linear pharmacokinetics, with exposure increasing in an approximately dose-proportional manner across the investigated range. The t1/2 and Vd remained relatively constant, with mean t1/2 values ranging, respectively, from 2.4 to 2.7 h and from 17 to 22 L (Table 3). Nacubactam was primarily eliminated via direct renal excretion, with renal clearance remaining consistent across doses. The majority of the administered dose was excreted unchanged in the urine, and metabolic clearance was minimal [106]. As with other BL/BLI combinations, the optimal PK/PD index for nacubactam is the percentage of fT > MIC. However, conventional PK/PD analyses may be challenging for BL/BLI combinations because the MIC of the β-lactam component varies with the concentration of the BLI. To address this issue, Igarashi et al. recently developed a novel PK/PD analysis method based on the concept of an instantaneous MIC (MICi), using different strains of β-lactamase-producing *Enterobacterales* [107]. This experimental study demonstrated that increasing nacubactam concentrations reduced cefepime MIC values in a concentration-dependent manner. In all tested strains, %fT > MICi showed a strong correlation with the mean change in bacterial load in a murine thigh infection model, suggesting that %fT > MICi is a robust PK/PD parameter for evaluating the cefepime/nacubactam combination. Specifically, the target %fT > MICi values required to achieve bacteriostatic activity, a 1-log_10_ reduction, and a 2-log_10_ reduction in bacterial counts were 30%, 49%, and 94%, respectively.

## 8. Discussion

The strategic repositioning of cefepime through combination with novel β-lactamase inhibitors reflects a broader strategy aimed at preserving the clinical value of a well-established β-lactam backbone in the context of escalating MDR and XDR Gram-negative resistance [1,2,15]. Despite its long-standing use, cefepime continues to offer favorable PK properties that support its applicability across a wide range of infection syndromes [3,24,26]. However, the global dissemination of ESBLs and carbapenemases has progressively undermined the reliability of cefepime monotherapy, prompting the development of adjunctive inhibitor-based strategies to restore and expand its antibacterial spectrum [14,36,37]. Importantly, the emerging cefepime-based combinations are far from homogeneous, differing substantially in microbiological coverage, PK/PD behavior, robustness of clinical evidence, and unresolved limitations.

From a microbiological standpoint, cefepime/enmetazobactam is primarily positioned to counter ESBL-mediated resistance through effective inhibition of class A β-lactamases, including CTX-M, TEM, and SHV enzymes (Table 4). This combination reliably restores cefepime activity against ESBL-producing *Enterobacterales* [2,33,41], but its spectrum remains limited. The absence of activity against MBLs, most class D OXA enzymes, and the majority of MDR *Pseudomonas aeruginosa* and *Acinetobacter baumannii* isolates [13,17,35] limits its clinical utility largely to infections caused by non-carbapenemase-producing *Enteobacterales*.

In contrast, cefepime/taniborbactam markedly broadens enzymatic coverage by inhibiting class A (including KPC), class C (AmpC), and class D (OXA-48-like) β-lactamases, while uniquely extending activity to selected MBLs such as NDM, VIM, and IMP variants [42,44,58,59]. This dual inhibition of serine β-lactamases and clinically relevant MBLs represents a conceptual advance over earlier inhibitors such as avibactam and relebactam [64,68,69,70,71,72]. Nevertheless, susceptibility remains enzyme-variant-dependent, and resistance has been documented in strains harboring NDM-9, NDM-30, and NDM-60 [14,40,55,56].

Cefepime/zidebactam introduces a mechanistically distinct paradigm by combining β-lactamase inhibition with high-affinity binding to PBP2, thereby acting as a “β-lactam enhancer” that complements cefepime’s preferential targeting of PBP3 [73,79,80,81,82]. This dual mechanism translates into potent in vitro activity against *Enterobacterales* and *Pseudomonas aeruginosa*, including isolates producing KPC, OXA-48, and MBLs, as well as strains resistant to ceftazidime/avibactam [80,81,82,83,84,85,97,103]. In addition, this strategy partially mitigates non-enzymatic resistance mechanisms such as efflux pump overexpression and porin loss [16,79]. However, reduced susceptibility associated with PBP2 or PBP3 alterations, particularly among NDM producers, raises concerns regarding the long-term robustness of this approach [73,79,85].

Cefepime/nacubactam occupies an intermediate position within this landscape. By combining potent inhibition of class A β-lactamases (including CTX-M and KPC) and class C AmpC enzymes with intrinsic PBP2 binding, nacubactam synergistically enhances cefepime activity [74,75,76,99,100,106,107]. This combination effectively restores susceptibility in many ESBL- and KPC-producing *Enterobacterales*. However, activity against class D enzymes and MBLs remains inconsistent, and efficacy against MDR *Pseudomonas aeruginosa* and *Acinetobacter baumannii* is limited [13,15,17].

From a clinical perspective, cefepime/enmetazobactam currently represents the most mature and best-validated combination. Phase III Integral-1 and Integral-2 trials demonstrated its non-inferiority to piperacillin/tazobactam in cUTI [33,41,42,43], and preclinical data indicating adequate pulmonary penetration support its potential applicability in pneumonia [42,46].

By contrast, cefepime/taniborbactam, despite offering the broadest theoretical enzymatic coverage, is supported by more limited clinical evidence. While encouraging results were observed in the phase III CERTAIN-1 trial for cUTI [33,41,42], the premature termination of CERTAIN-2 (designed to evaluate HAP and VAP vs. meropenem) significantly constrains confidence in its role for severe systemic infections [62,63]. This uncertainty is further compounded by regulatory challenges, exemplified by the FDA’s 2024 decision not to approve the New Drug Application [33].

Cefepime/zidebactam and cefepime/nacubactam remain earlier in their development trajectories. Evidence for cefepime/zidebactam is currently derived primarily from phase I studies, PK/PD modeling, and limited compassionate-use experience [83,90,91,92,93,94,95], while cefepime/nacubactam, despite being supported by a strong microbiological rationale and innovative PK/PD (e.g., instantaneous MIC), lacks definitive clinical efficacy data, with pivotal trials still ongoing [106,107].

Across all cefepime-based combinations, several shared limitations warrant consideration. Non-enzymatic resistance mechanisms, including porin loss, efflux pump overexpression, and PBP alterations, remain clinically relevant and may compromise activity irrespective of β-lactamase inhibition [12,16,18]. Furthermore, pivotal trials have consistently under-represented critically ill populations and geographic regions with a high prevalence of MBL- or OXA-type carbapenemases [13,40,41]. Trial discontinuations, such as CERTAIN-2, further limit the available evidence base for severe infections [62,63]. Finally, regulatory and stewardship-related constraints represent a non-trivial barrier to approval, implementation, and widespread clinical adoption [33,45,57,69].

## 9. Conclusions

At present, cefepime/enmetazobactam has the most clearly defined clinical role, supported by phase III evidence in infections caused by ESBL-producing Enterobacterales. Broader-spectrum combinations such as cefepime/taniborbactam and cefepime/zidebactam offer compelling mechanistic and microbiological advantages but remain constrained by incomplete clinical validation in severe infections and ongoing regulatory uncertainty. Cefepime/nacubactam occupies an intermediate position, underpinned by strong microbiological and PK/PD rationale yet still lacking definitive outcome data. Ultimately, the clinical impact of cefepime-based combinations will depend on the resolution of key evidence gaps, particularly in infections driven by MBL-producing organisms, without which their benefit is likely to remain context-dependent and potentially time-limited.

## Figures and Tables

**Table 1:** Cefepime breakpoints (valid from 1 January 2025).

	MIC (mg/L)	
CATEGORY	CLSI	EUCAST
*Enterobacterales* ^1^		
Susceptible	≤2	≤1
SDD (CLSI)	4–8	
Resistant	≥16	>4
*P. aeruginosa*		
Susceptible	≤8	≤0.001
I^ (CLSI)	16	
Resistant	≥32	>8

^1^ CLSI MIC breakpoints for *Enterobacterales* (excluding *Salmonella* and *Shigella* spp.). I^ potential to concentrate in the urine.

**Table 2:** Antimicrobial activity (MIC_50_ and MIC_90_) of cefepime with and without a β-lactamase inhibitor.

Antibiotics	*K. pneumoniae*		*E. coli*		*P. aeruginosa*		*A. baumannii*	
	MIC_50_	MIC_90_	MIC_50_	MIC_90_	MIC_50_	MIC_90_	MIC_50_	MIC_90_
Cefepime	0.06	256	0.06	32	4	32	32	128
Cefepime/enmetazobactam	0.03	0.25	0.03	0.125	2	16	0.08	64
Cefepime/taniborbactam	NA	NA	NA	NA	NA	NA	16	64
Cefepime/zidebactam	0.06	0.5	0.03	0.125	1	4	NA	NA
Cefepime/nacubactam	NA	NA	NA	NA	NA	NA	NA	NA

MIC_50_ and MIC_90_ values extracted from EUCAST epidemiological cutoff (ECOFF) MIC distributions as of January 2026. NA (not available) indicates insufficiency of international surveillance data meeting EUCAST criteria (≥100 total isolates from ≥5 independent datasets with consistent modal MICs) and does not reflect lack of in vitro activity.

**Table 3:** Main pharmacokinetic parameters of cefepime and the four beta-lactamase inhibitors under development.

PK Parameter	Cefepime	Enmetazobactam	Nacubactam	Taniborbactam	Zidebactam
Dosing ratio	-	4:1	2:1	4:1	2:1
Tested doses °	2 g q8h	0.5 g q8h	1 g q8h	0.5 g q8h	1 g q8h
Vd, L	18–23	21	17–22	20	15–20
Half-life, h	2	2.6	1.8–2.4	2.3–5.8	2
Protein binding, %	20%	<1%	4%	<1%	15%
Renal elimination, %	85%	90%	90%	90%	>80%
ELF/plasma ratio, %	20–60%	30–75%	30% *	15–25%	35–38%

* Data from mice. ELF: epithelial lining fluid; Vd: volume of distribution. ° In patients with normal renal function.

**Table 4:** Microbiological targets and clinical development status of cefepime and its β-lactam/β-lactamase inhibitor combinations.

Agent/Combination	Inhibitor Class/Mechanism	Key β-Lactamases Inhibited	Principal Microbiological Targets	Activity Against Carbapenemase Producers	Clinical Development (Trials/Status)	Key Findings/Comments
Cefepime	/	Limited inhibition; not active vs. ESBLs or carbapenemases	*Enterobacterales* (non-ESBL), *P. aeruginosa*	None	Approved; extensive clinical use since 1990s	Active vs. AmpC producers; good CNS penetration; neurotoxicity risk in renal impairment
Cefepime/enmetazobactam	DBO β-lactamase inhibitor	Class A ESBLs (TEM, SHV, CTX-M), some class C	*ESBL-producing* *Enterobacterales*	Partial (KPC only)	Phase III (NCT03687255, completed)	Demonstrated non-inferiority to piperacillin/tazobactam in cUTI; favorable PK/PD and safety
Cefepime/taniborbactam	Cyclic boronate β-lactamase inhibitor	Class A (KPC, ESBL), C (AmpC), D (OXA-48-like), selected B (NDM, VIM)	*Enterobacterales*, *P. aeruginosa*	Broad, including some MBLs	Phase III (CERTAIN-1, completed; CERTAIN-2, suspended)	Superior to meropenem in cUTI; effective against MBL-producing isolates; well tolerated
Cefepime/zidebactam	DBO; dual β-lactamase inhibition + PBP2 binding	Class A, C; limited D; indirect effect vs. MBLs	*Enterobacterales*, *P. aeruginosa*, *A. baumannii*	Partial (via PBP2 mechanism)	Phase III (NCT04979806, completed)	Well tolerated in renal function studies; real-world Indian case series show efficacy vs. NDM-*P. aeruginosa*
Cefepime/nacubactam	DBO; β-lactamase inhibition + PBP2 binding	Class A (KPC, ESBL), C (AmpC)	*Enterobacterales*, some *P. aeruginosa*, *A. baumannii*	Limited (KPC only)	Phase III (Integral-1, completed; Integral-2, ongoing)	Active vs. CRE; results pending from cUTI and HAP/VAP trials; promising carbapenem-sparing potential

Abbreviations: AmpC, class C β-lactamase; CRE, carbapenem-resistant *Enterobacterales*; DBO, diazabicyclooctane β-lactamase inhibitor; ESBL, extended-spectrum β-lactamase; KPC, *Klebsiella pneumoniae* carbapenemase; MBL, metallo-β-lactamase; OXA, oxacillinase; PBP2, penicillin-binding protein 2; cUTI, complicated urinary tract infection; HAP/VAP, hospital-acquired or ventilator-associated pneumonia.

## Data Availability

No new data were created or analyzed in this study.
